# Supplementary material for: UtpA and UtpB chaperone nascent pre-ribosomal RNA and U3 snoRNA to initiate eukaryotic ribosome assembly
Source: Nat Commun. 2016 Jun 29;7:12090. doi: 10.1038/ncomms12090 (PMC4931317; doi:10.1038/ncomms12090)
Supplement: Supplementary Figures and References — Supplementary Figures 1-11 and Supplementary References [file ncomms12090-s1.pdf]

Supplementary Information for

UtpA and UtpB chaperone nascent pre-ribosomal RNA and U3 snoRNA to initiate eukaryotic ribosome assembly

Mirjam Hunziker[1,7], Jonas Barandun[1,7], Elisabeth Petfalski[2], Dongyan Tan[3], Clémentine Delan-Forino[2], Kelly R. Molloy[4], Kelly H. Kim[5], Hywel Dunn-Davies[2], Yi Shi[4], Malik Chaker-Margot[1,6], Brian T. Chait[4], Thomas Walz[5], David Tollervey[2] and Sebastian Klinge[1,*]

[1] Laboratory of Protein and Nucleic Acid Chemistry, The Rockefeller University, New York, New York, USA

[2] Wellcome Trust Centre for Cell Biology, University of Edinburgh, Edinburgh, Scotland

[3] Department of Cell Biology, Harvard Medical School, Boston, MA, USA

[4] Laboratory of Mass Spectrometry and Gaseous Ion Chemistry, The Rockefeller University, New York, USA

[5] Laboratory of Molecular Electron Microscopy, The Rockefeller University New York, New York, USA

[6] Tri-Institutional Training Program in Chemical Biology, The Rockefeller University, New York, New York, USA

[7] Contributed equally.

[*] Correspondence should be addressed to S.K. (klinge@rockefeller.edu).

This PDF file includes:

Supplementary Figs. 1-11
Supplementary References 1-8

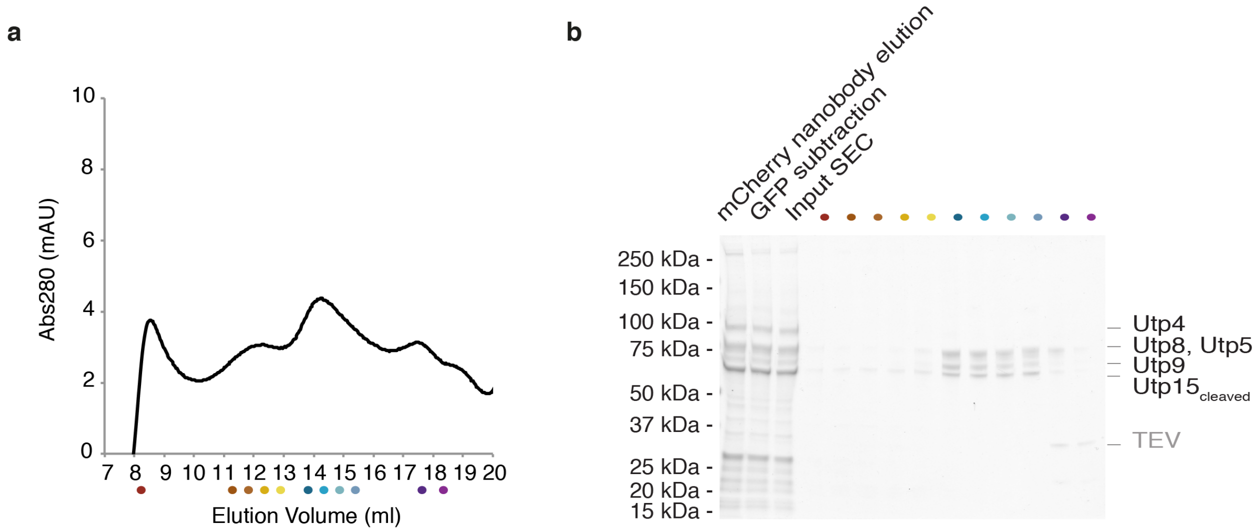

Supplementary Figure 1. Utp4 is loosely associated with the tetrameric subcomplex consisting of Utp5, Utp8, Utp9 and Utp15. **(a)** Size-exclusion chromatogram of a purified pentameric UtpA subcomplex consisting of Utp4, Utp5, Utp8, Utp9 and Utp15-3myc. **(b)** Visualization of the purification steps. First capture of overexpressed Utp15-3myc-TEV-mCherry and associated subunits by anti-mCherry sepharose followed by TEV protease elution (lane 1, mCherry nanobody elution), subtractive removal of Utp10-GFP containing endogenous UtpA (lane 2, GFP subtraction), the input for size exclusion chromatography (lane 3, input SEC) and color coded peak fractions are shown on a Coomassie-blue stained 4-12% SDS-PAGE gel.

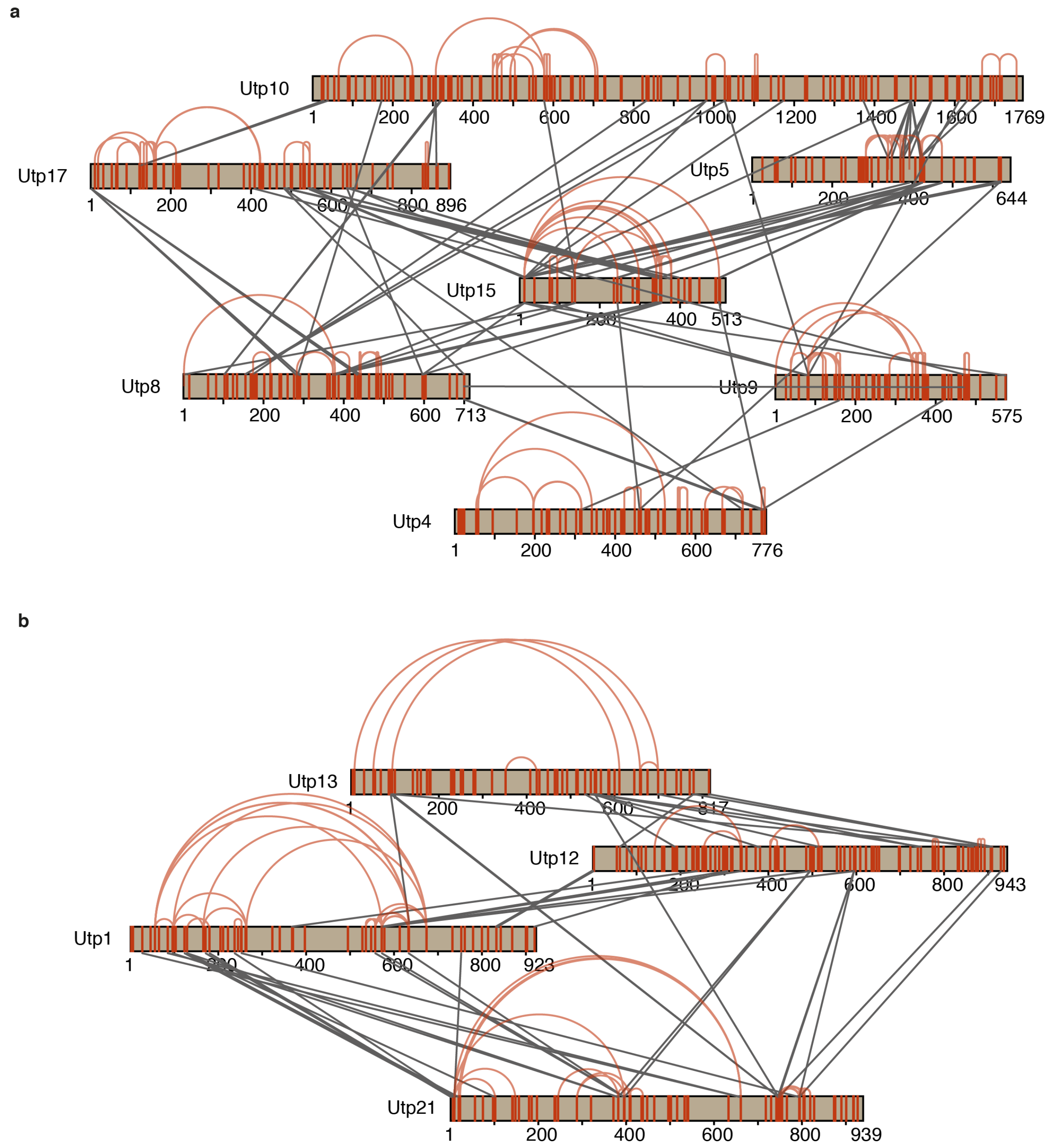

Supplementary Figure 2. Visualization of DSS cross-linking and mass spectrometry analysis of *Saccharomyces cerevisiae* UtpA **(a)** and UtpB lacking Utp6 and Utp18 **(b)**. Subunits of UtpA and UtpB are shown in grey with numbered amino acids indicated. Inter-subunit and intra-subunit cross-links are depicted as dark-grey and red lines, respectively. Lysine positions are shown in red within subunits. This figure was prepared using xiNet[1].

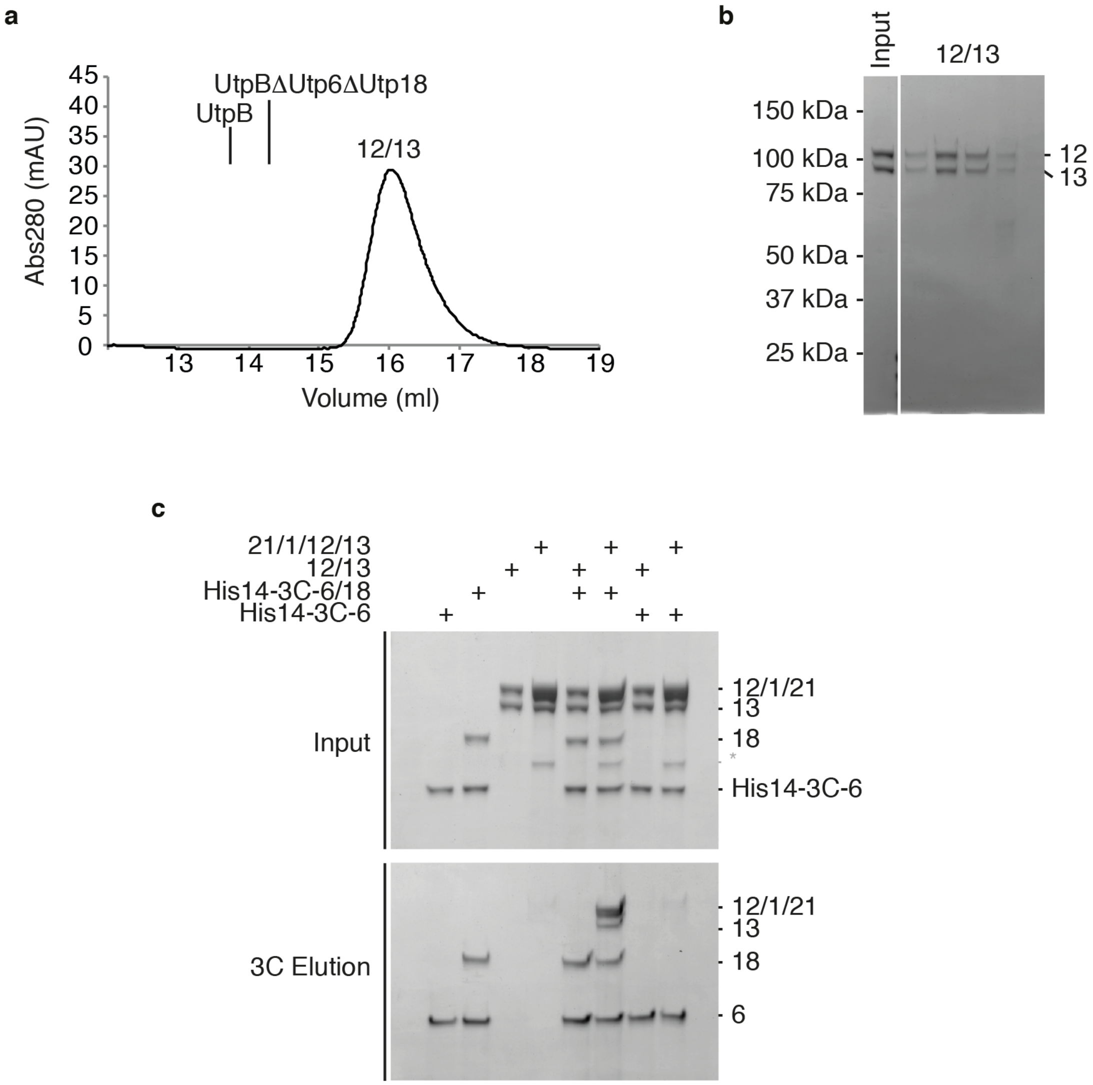

Supplementary Figure 3. Utp12 and Utp13 form a stoichiometric complex and require Utp21/Utp1 to interact with Utp6/Utp18. (**a**) Size-exclusion chromatogram of the heterodimer Utp12/Utp13 (black line). Elution volumes of UtpB and UtpB lacking Utp6 and Utp18 are indicated for comparison. (**b**) Visualization of the main peak fractions from (a) by 4-12% SDS-PAGE. (**c**) Pull-down assay performed using Nickel sepharose beads and monomeric His-tagged Utp6 or heterodimeric His-tagged Utp6/Utp18 as bait. The upper gel shows pre-incubated input samples, the lower gel the elution of bound proteins by 3C protease cleavage. The asterisk indicates a GroEL contamination. Samples were analyzed by 4-12% SDS-PAGE and Coomassie-blue staining.

a

b

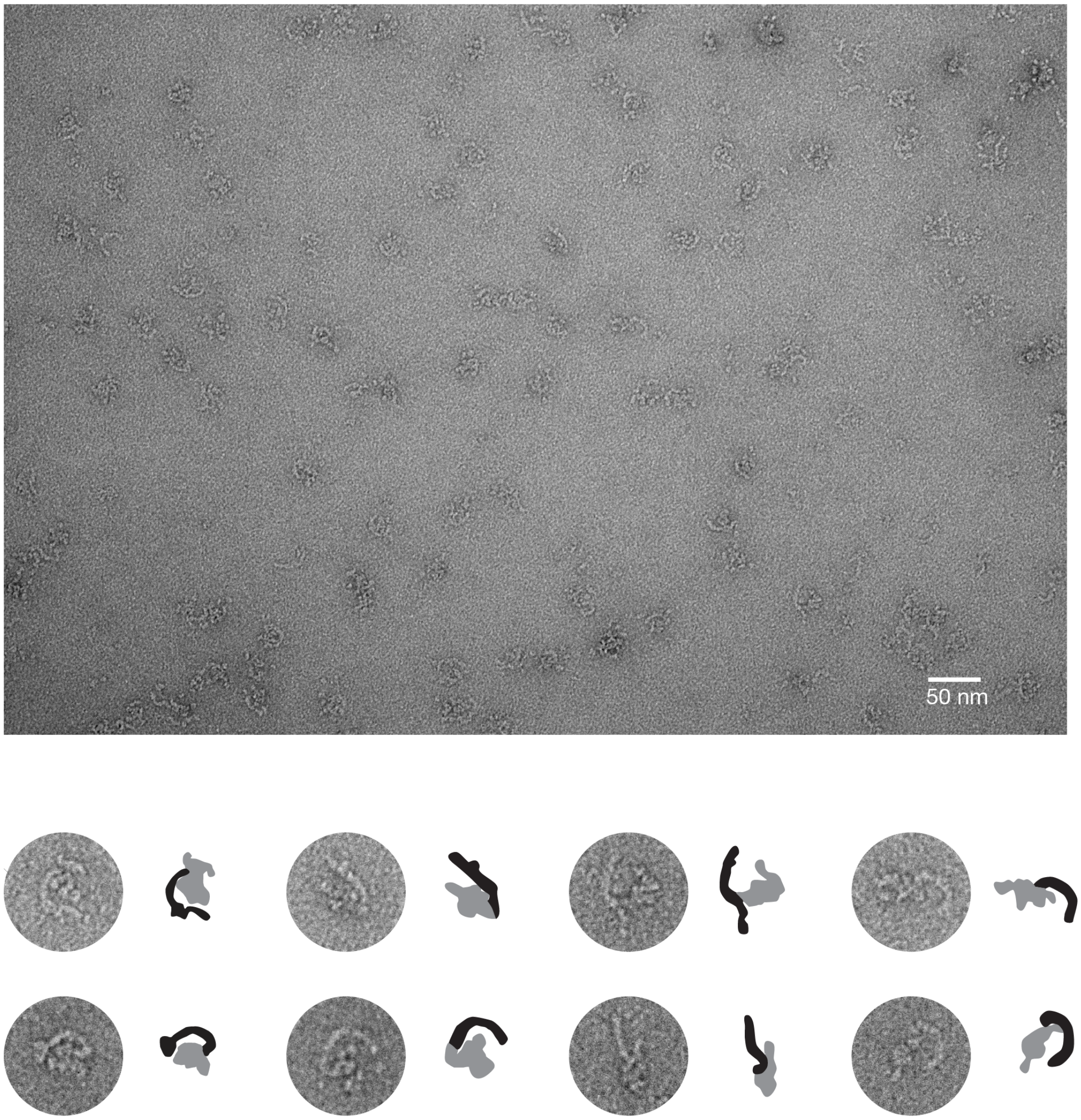

Supplementary Figure 4. Visualization of the UtpA complex by negative-stain EM. (**a**) A representative micrograph with a scale bar on the bottom right. (**b**) Gallery of representative particles showing that UtpA can adopt a wide variety of conformations. Outlines of the particles were traced and are displayed as schematics on the right of each particle. An elongated feature is colored in black, while the body is colored in grey. The diameter of the circle is 56 nm.

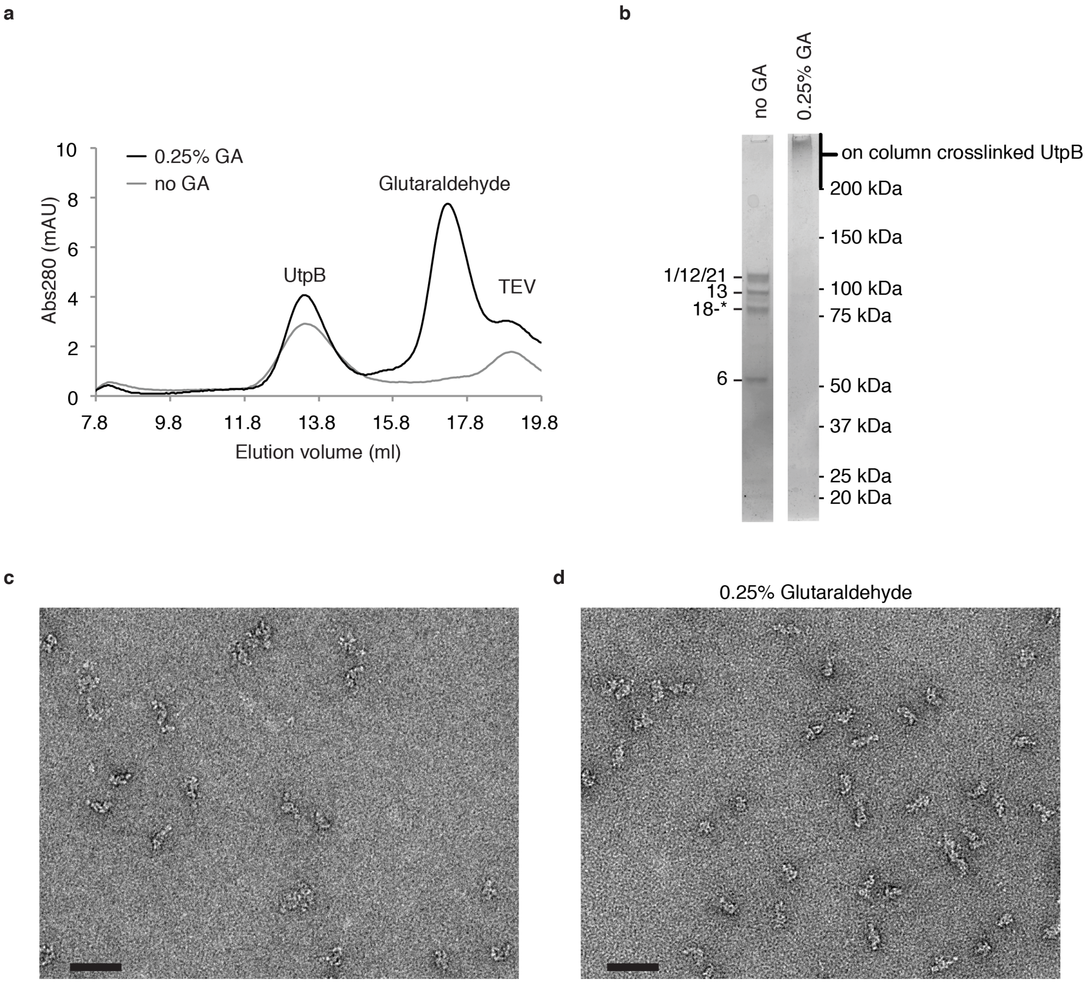

Supplementary Figure 5. On-column glutaraldehyde cross-linking of *Saccharomyces cerevisiae* UtpB for structural studies. (**a**) Representative size-exclusion chromatogram of UtpB in the presence (black) or absence (grey) of a pre-injected glutaraldehyde bolus as previously described[2]. (**b**) Visualization of both main peak fractions from (a) by 4-12% SDS-PAGE showing complete cross-linking of UtpB in the presence of 0.25% glutaraldehyde. The asterisk indicates that Utp18-3myc-TEV-mCherry-Flag was used as affinity purification tag and Utp18 contains three C-terminal myc tags after TEV cleavage. (**c, d**) Representative areas of EM images of negatively stained endogenous UtpB that was not cross-linked (c) or on-column cross-linked with 0.25% glutaraldehyde (d). Scale bars are 50 nm.

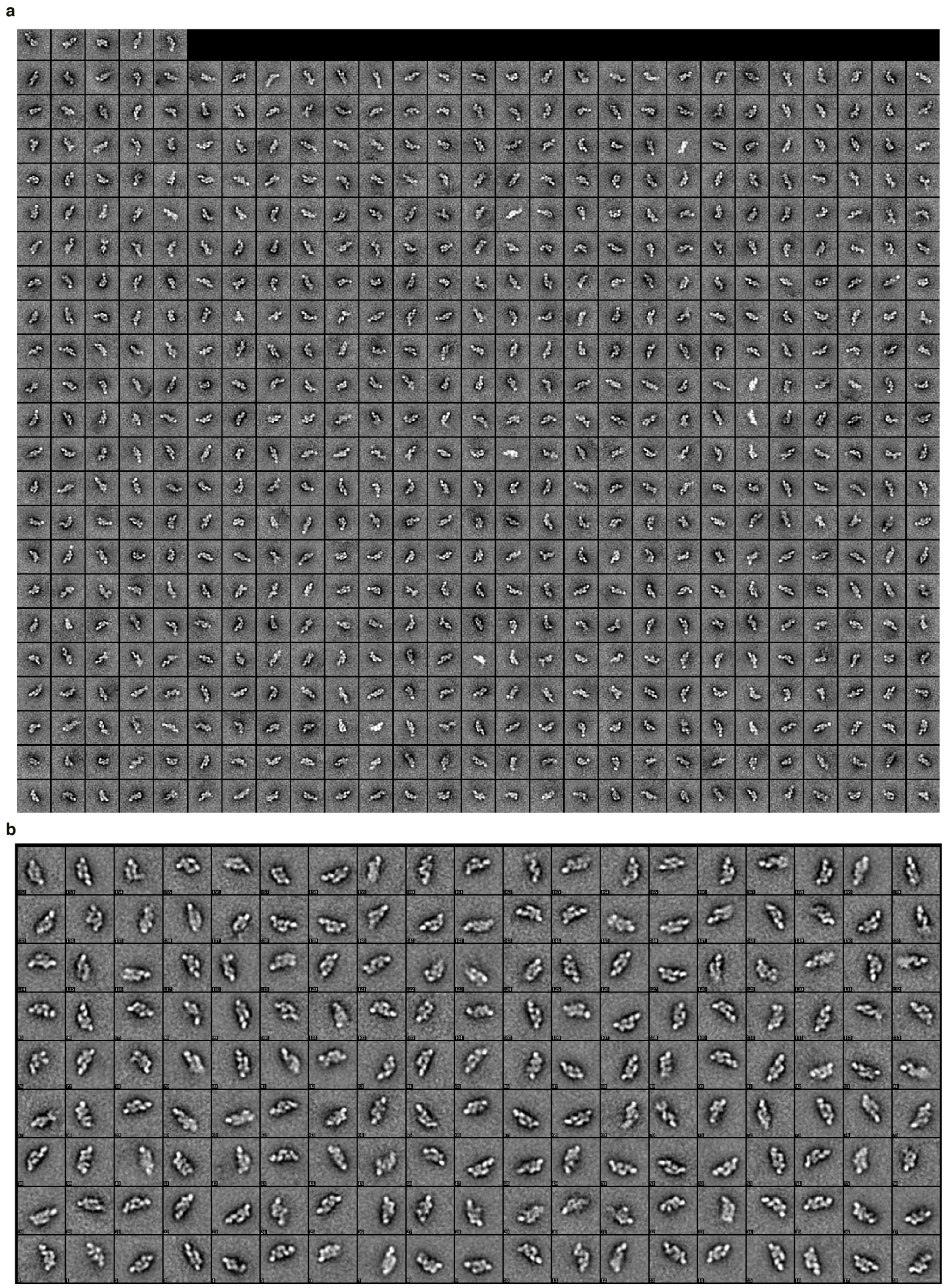

Supplementary Figure 6. ISAC[3] 2D class averages of endogenous UtpB and recombinant UtpB lacking Utp6 and Utp18. (**a**) 598 class averages of negatively stained and cross-linked endogenous UtpB obtained by ISAC. (**b**) 171 class averages of negatively stained and cross-linked reconstituted UtpB lacking Utp6 and Utp18 obtained by ISAC. The side length of the individual panels is 500 Å in (a) and 364 Å in (b).

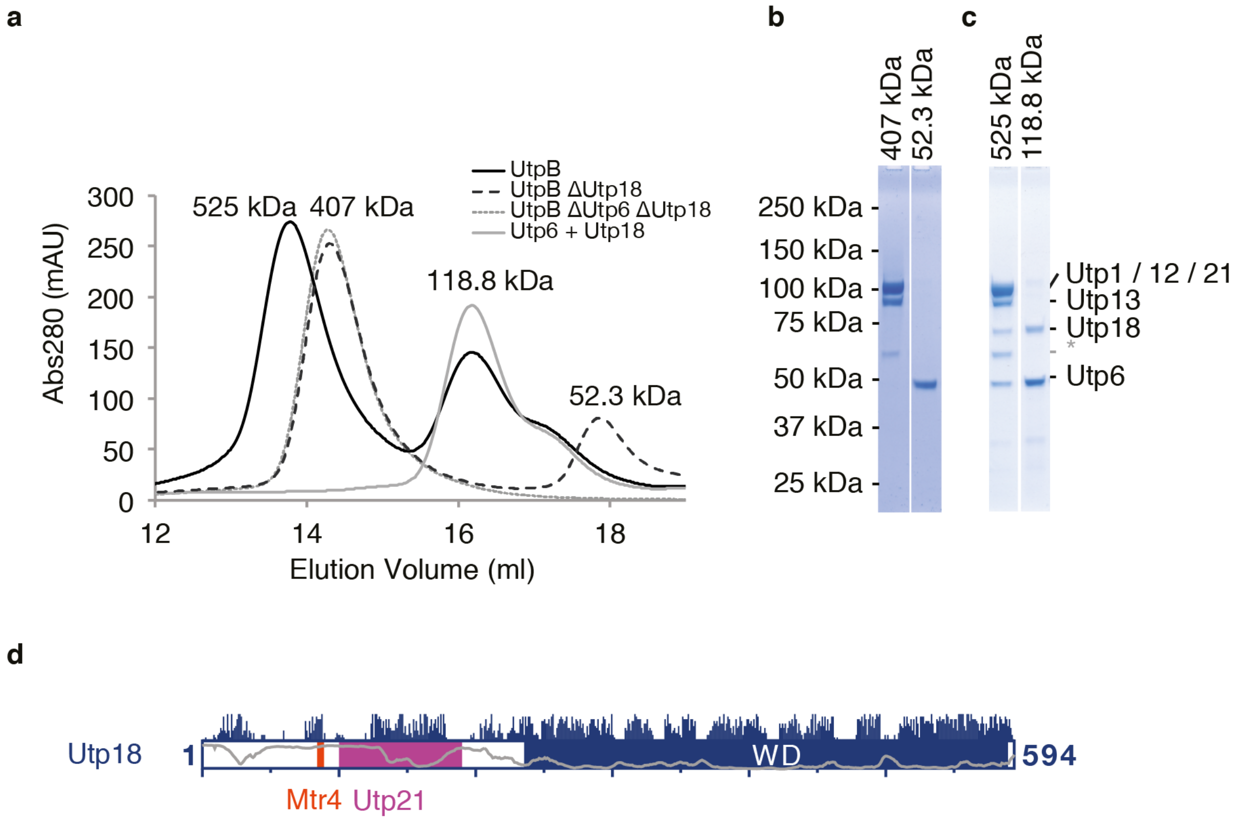

Supplementary Figure 7. Utp6 requires Utp18 to interact with the tetrameric core of UtpB. (**a**) Size-exclusion chromatograms of heteropentameric UtpB lacking Utp18 (black dashed line), heterotetrameric UtpB (lacking Utp6 and Utp18, grey dotted line), heterohexameric UtpB (black line) and heterodimeric Utp6/Utp18 (grey line). Predicted molecular sizes are indicated on top of the corresponding peaks. (**b, c**) Corresponding 4-12% SDS-PAGE analysis of the main peak fractions shown in (a). The gel in (b) shows the main peak fractions of heteropentameric UtpB lacking Utp18 [black dashed line in (a)] while the gel in (c) shows the main peak fractions of heterohexameric UtpB [black line in (a)]. The asterisk indicates a GroEL contamination. (**d**) Schematic representation of Utp18 showing the C-terminal WD40 domain in blue and interaction motifs for Mtr4 and Utp21 in red and pink, respectively. Conservation is plotted on top of the schematic and disorder disposition is indicated by a PONDR-Fit plot[4] (grey line) with regions with intrinsic disorder (disorder disposition > 0.5) and more ordered regions (disorder disposition < 0.5).

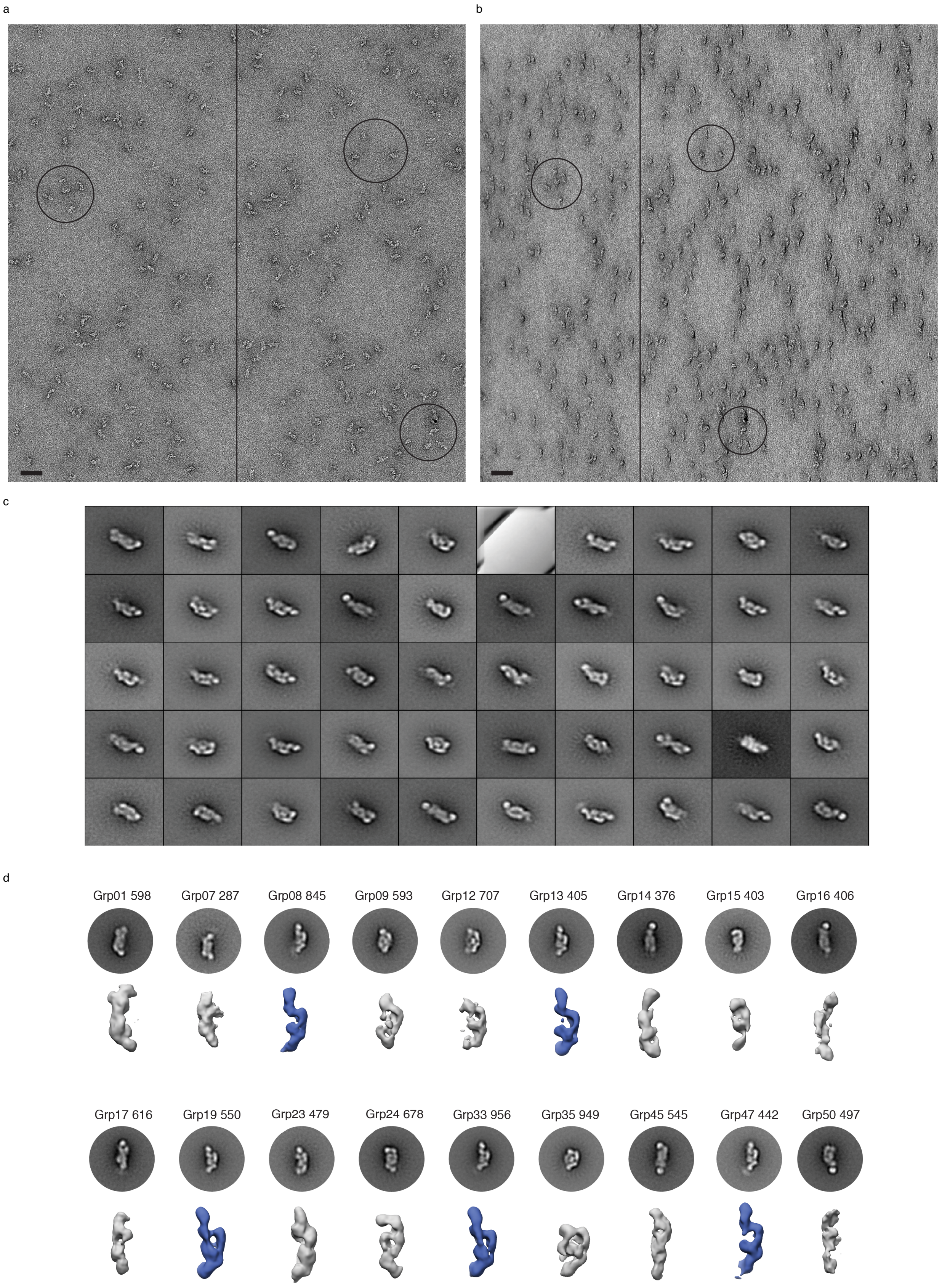
a
b
c
d
Grp01 598
Grp07 287
Grp08 845
Grp09 593
Grp12 707
Grp13 405
Grp14 376
Grp15 403
Grp16 406
Grp17 616
Grp19 550
Grp23 479
Grp24 678
Grp33 956
Grp35 949
Grp45 545
Grp47 442
Grp50 497

Supplementary Figure 8. Random conical tilt 3D reconstruction of endogenous *Saccharomyces cerevisiae* UtpB. (**a, b**) Representative images of the same specimen area of negatively stained endogenous UtpB recorded at tilt angles of 0º (a) and 60º (b). Examples of corresponding areas in the two images are circled. The black line indicates the tilt axis. The scale bar represents 50 nm. (**c**) 2D class averages from 10 cycles of multi-reference alignment using SPIDER[5]. Each round of multi-reference alignment was followed by K-means classification specifying 50 output classes. The side length of each panel is 50 nm. (**d**) Representative set of class averages. The group number and number of particles within each group are indicated on the top of the 2D class average and the corresponding 3D reconstruction is shown below. Volumes from 8 iterations of back-projection refinement are shown. All reconstructions were low-pass filtered to 20 Å resolution. Reconstructions that were combined and subjected to angular refinement are colored in blue.

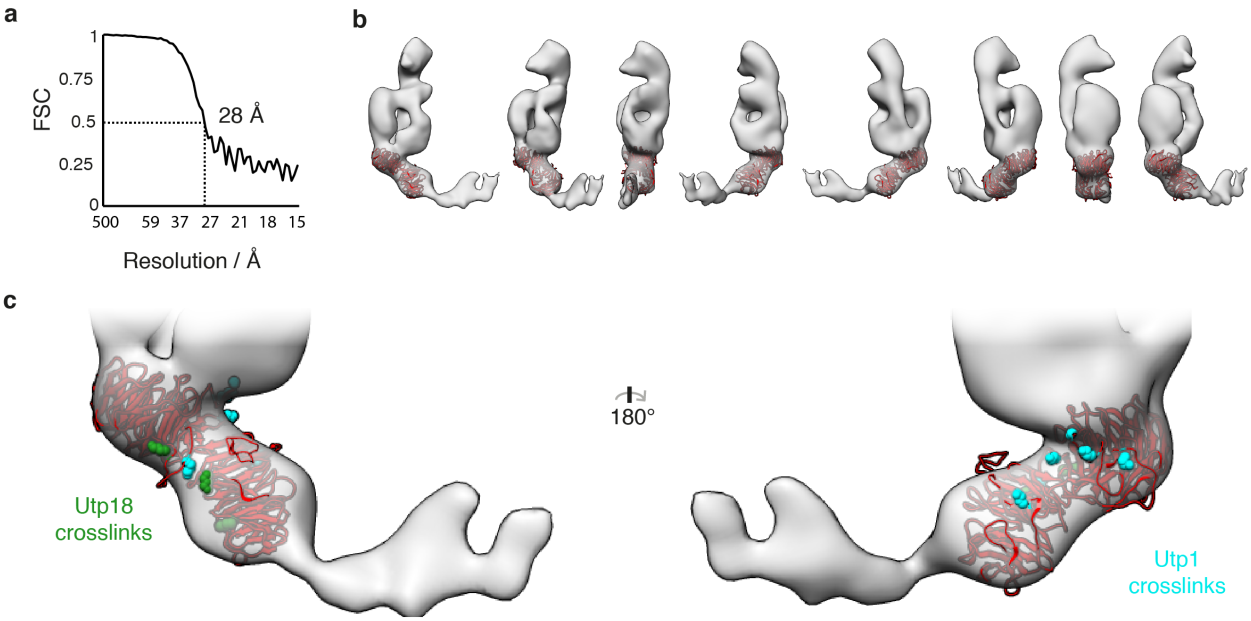

Supplementary Figure 9. 3D density map of UtpB and docking of the crystal structure of Utp21. (**a**) Fourier shell correlation (FSC) curve for the final 3D density map estimates a resolution of 28 Å using an FSC cut-off of 0.5. (**b**) Volume of UtpB obtained by combining several classes (Supplementary Figure 8d: blue volumes, grp8, 13, 19, 33 and 47) and using angular refinement in SPIDER. The crystal structure of the tandem β-propeller of Utp21[6] is shown in red (pdb code 4nsx). (**c**) Zoomed in view on the lower foot region of UtpB. Weak density can be observed for the flexible Utp6/Utp18 heterodimer. A model of the Utp21 tandem β-propeller was generated to include missing loops not resolved in the crystal structure using MODELLER[7] and pdb code 4nsx as template. Lysines (shown as spheres) cross-linked to Utp1 are colored in cyan while lysines cross-linked to Utp18 are shown in green.

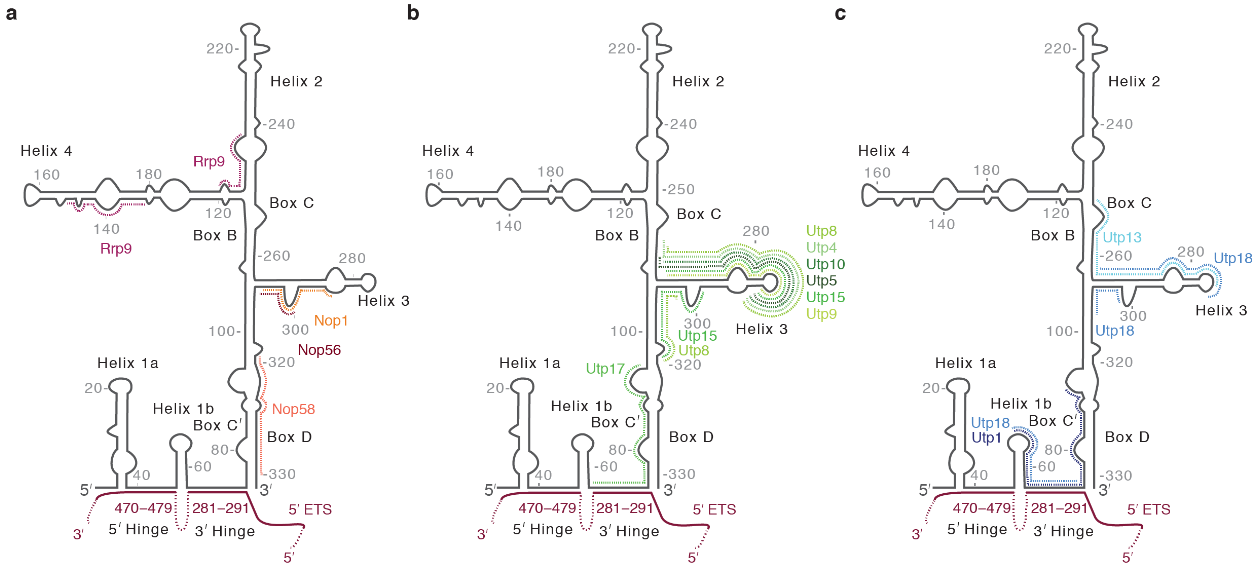

Supplementary Figure 10. UtpA and UtpB contact the U3 snoRNA. (**a, b, c**) Schematic secondary structure of U3 snoRNA (black) with base-paired 5´ ETS (purple) with CRAC-based binding sites for the U3 snoRNP proteins Rrp9, Nop1, Nop56 and Nop58 as determined previously[8] (a), all UtpA subunits (b) and selected UtpB subunits (c). For each subunit nucleotide positions with the highest frequency of recovery (top 10%) were plotted on the secondary structure of the U3 snoRNA.

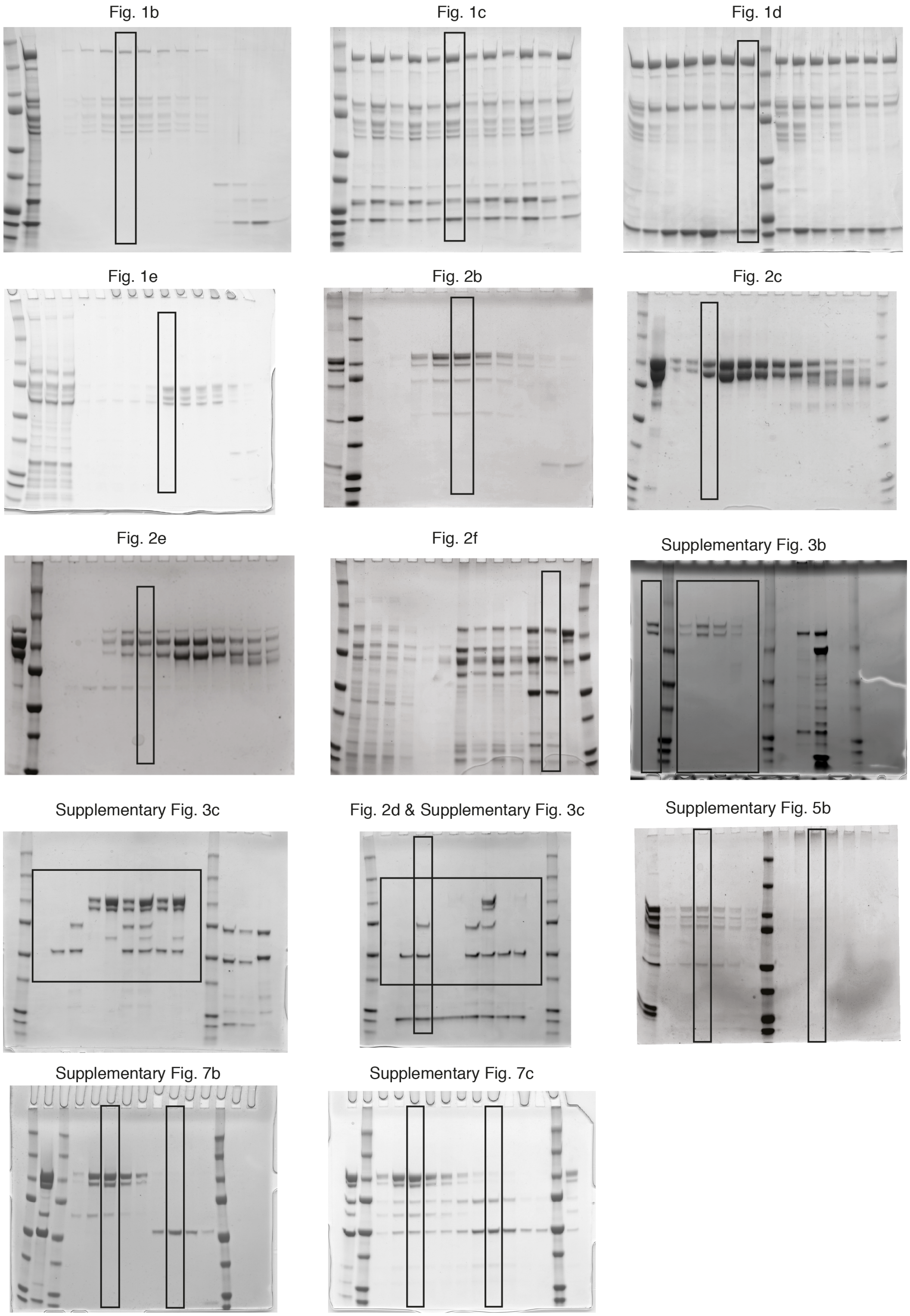

Supplementary Figure 11. Uncut raw SDS-PAGE gels. Black boxes indicate cropped regions that were used in the main manuscript or supplementary information.

References

1. Combe, C. W., Fischer, L. & Rappsilber, J. xiNET: cross-link network maps with residue resolution. *Mol. Cell Proteomics* **14,** 1137–1147 (2015).
2. Shukla, A. K. *et al.* Visualization of arrestin recruitment by a G-protein-coupled receptor. *Nature* **512,** 218–222 (2014).
3. Yang, Z., Fang, J., Chittuluru, J., Asturias, F. J. & Penczek, P. A. Iterative stable alignment and clustering of 2D transmission electron microscope images. *Structure* **20,** 237–247 (2012).
4. Xue, B., Dunbrack, R. L., Williams, R. W., Dunker, A. K. & Uversky, V. N. PONDR-FIT: a meta-predictor of intrinsically disordered amino acids. *Biochim. Biophys. Acta* **1804,** 996–1010 (2010).
5. Frank, J. *et al.* SPIDER and WEB: processing and visualization of images in 3D electron microscopy and related fields. *J. Struct. Biol.* **116,** 190–199 (1996).
6. Zhang, C. *et al.* Structure of Utp21 tandem WD domain provides insight into the organization of the UTPB complex involved in ribosome synthesis. *PLoS ONE* **9,** e86540 (2014).
7. Webb, B. & Sali, A. Comparative Protein Structure Modeling Using MODELLER. *Curr Protoc Bioinformatics* **47,** 5.6.1–32 (2014).
8. Granneman, S., Kudla, G., Petfalski, E. & Tollervey, D. Identification of protein binding sites on U3 snoRNA and pre-rRNA by UV cross-linking and high-throughput analysis of cDNAs. *Proc. Natl. Acad. Sci. U.S.A.* **106,** 9613–9618 (2009).
